# Assessment of whole blood thrombosis in a microfluidic device lined by fixed human endothelium

**DOI:** 10.1007/s10544-016-0095-6

**Published:** 2016-07-27

**Authors:** Abhishek Jain, Andries D. van der Meer, Anne-Laure Papa, Riccardo Barrile, Angela Lai, Benjamin L. Schlechter, Monicah A. Otieno, Calvert S. Louden, Geraldine A. Hamilton, Alan D. Michelson, Andrew L. Frelinger, Donald E. Ingber

**Affiliations:** 1 Wyss Institute for Biologically Inspired Engineering, Harvard University, 3 Blackfan Circle, CLSB 5, Boston, MA 02115 USA; 2 Division of Hemostasis and Thrombosis, Department of Medicine, Beth Israel Deaconess Medical Center, Harvard Medical School, Boston, MA USA; 3 Vascular Biology Program and Department of Surgery, Boston Children’s Hospital and Harvard Medical School, Boston, MA USA; 4 MIRA Institute for Biomedical Technology and Technical Medicine, University of Twente, Enschede, The Netherlands; 5 Board of Governors Regenerative Medicine Institute, Cedars-Sinai Medical Center, Los Angeles, CA USA; 6 Department of Biomedical Engineering, Carnegie Mellon University, Pittsburgh, PA USA; 7 Division of Hematology and Oncology, Department of Medicine, Beth Israel Deaconess Medical Center, Harvard Medical School, Boston, MA USA; 8 Janssen Pharmaceutical Research and Development, Pre-Clinical Development and Safety, Spring House, PA USA; 9 Emulate Inc., 210 Broadway St., Cambridge, MA USA; 10 Center for Platelet Research Studies, Division of Hematology/Oncology, Boston Children’s Hospital, Dana-Farber Cancer Institute, Harvard Medical School, Boston, MA USA; 11 Harvard John A. Paulson School of Engineering and Applied Sciences, Cambridge, MA USA

**Keywords:** Vascular endothelium, Hemostasis, Thrombosis, Biomedical technology, Platelet function tests, Lab-on-a-Chip

## Abstract

The vascular endothelium and shear stress are critical determinants of physiological hemostasis and platelet function *in vivo*, yet current diagnostic and monitoring devices do not fully incorporate endothelial function under flow in their assessment and, therefore, they can be unreliable and inaccurate. It is challenging to include the endothelium in assays for clinical laboratories or point-of-care settings because living cell cultures are not sufficiently robust. Here, we describe a microfluidic device that is lined by a human endothelium that is chemically fixed, but still retains its ability to modulate hemostasis under continuous flow *in vitro* even after few days of storage. This device lined with a fixed endothelium supports formation of platelet-rich thrombi in the presence of physiological shear, similar to a living arterial vessel. We demonstrate the potential clinical value of this device by showing that thrombus formation and platelet function can be measured within minutes using a small volume (0.5 mL) of whole blood taken from subjects receiving antiplatelet medications. The inclusion of a fixed endothelial microvessel will lead to biomimetic analytical devices that can potentially be used for diagnostics and point-of-care applications.

**Electronic supplementary material:**

The online version of this article (doi:10.1007/s10544-016-0095-6) contains supplementary material, which is available to authorized users.

## Introduction

Mutual signaling between vascular endothelium and platelets is critical for regulation of haemostasis and thrombotic disorders associated with various diseases. (Alexandru et al. 2012; Lowenberg et al. 2010; Siddiqui et al. 2013; Wu and Thiagarajan 1996) Yet no practical diagnostic assays exist that can measure cross-talk between platelets and inflamed vessel walls in the presence of physiological shear. Over the last decade, microfluidic devices that contain hollow channels lined by living endothelium have been exposed to flowing blood to study the basic science of thrombosis *in vitro* (Colace et al. 2013; Neeves et al. 2013; Tsai et al. 2012; Zheng et al. 2012). While these devices are useful for advancing research, they are not used in practical or clinical settings because living cell cultures are not robust and the devices can not be stored for extended times. The rare microfluidic devices that have been used in diagnostic settings are typically lined with collagen to mimic thrombus formation and platelet aggregation induced in response to vascular wall injury. While more relevant than coagulation assays carried out in solution, these devices still fail to capture the dynamic physiological interplay between endothelial cells, platelets and fluid shear stress that are critical for control of blood clotting in inflammatory diseases (Branchford et al. 2015; Neeves et al. 2013).

The aim of this study was to explore whether a microfluidic device lined by a chemically preserved (fixed) human endothelium will retain its ability to support thrombus formation and platelet adhesion when human whole blood is flowed through the channel at an arterial shear rate. If this were possible, it could provide a way to create more robust devices for analysis of haemostasis and thrombosis under more physiologically relevant conditions in clinical laboratories and point-of-care settings in the future. Here, we demonstrate that microfluidic devices lined by fixed human endothelium can be used in this manner, and we demonstrate its physiological relevance by comparing results obtained from analysis of blood samples from human patients taking antiplatelet medication with results obtained using standard aggregometry or similar collagen-coated microfluidic devices.

## Materials and methods

### Microfluidic device design and fabrication

The microfluidic device was designed using AutoCAD software, master templates were fabricated by photolithography on Si (100) wafers (University Wafer Corp.) and soft lithography was used to prepare the devices with polydimethylsiloxane (PDMS) (Duffy et al. 1998). We placed six independent microchannels (each 400 μm wide, 100 μm high, 2 cm long) on the PDMS block with the dimensions of a standard glass slide (75 × 25 mm). The inlet well was cut to a size of 3.5 mm with a punch (Harris Uni Core) in order to fit an open slip-tip syringe head (BD Biosciences) that was used as a blood reservoir. The outlet was punched to a size of 1 mm. A 1/16″ female barbed luer connector was inserted in the outlet and 1/16″ ID medical grade tygon tubing (Formula: ND-100-05) of 35 cm was inserted in this connector. The other end of the tubing was connected to a 3 mL syringe that was fitted to a syringe pump (Harvard Apparatus, PhD Ultra).

### Microchannel functionalization, endothelial culture, fixation and fluorescent staining

The microfluidic devices were exposed to oxygen plasma for 30 s, at a power of 50 W, using a PE-100 plasma sterilizer (Plasma Etch, Inc. NV, USA) and then treated with 1 % (3-aminopropyl)-trimethoxysilane (APTMS; Sigma) in 100 % anhydrous ethanol, for 10 min. After subsequent rinsing with 70 % ethanol and 100 % ethanol, the devices were baked at 80 °C for 2 h. Then, the inside of the channels was coated with rat type I collagen (100 μg/mL; Corning) overnight at 37 °C in a 5 % CO_2_ incubator, followed by rinsing with Endothelial Growth Medium-2 (EGM-2; Lonza). To line all four sides of the rectangular channel with endothelium, human umbilical vein endothelial cells (HUVECs; mixed donor; 1.25 × 10^7^/mL; Lonza) were first flowed into the collagen-coated microchannels. Then, the devices were incubated for 20 min while upside down, after which a fresh HUVEC suspension was introduced in the channels and incubated for eight additional hours to promote cell attachment and spreading on all surfaces of the channel. The channels were then rinsed with EGM-2, with or without TNF-α (recombinant from *E. coli*, Sigma). After incubating for up to 18 h, a 4 % formaldehyde solution (Sigma) was flushed through the channels and the devices were incubated for 15 min at room temperature. Finally, the devices were rinsed twice with EGM-2 and then placed at 4 °C for upto 36 h before use. Mouse or rabbit antibodies against intercellular adhesion molecule-1 (ICAM-1), vascular adhesion molecule-1 (VCAM-1), tissue factor (TF, Santa Cruz), von Willebrand Factor (VWF, Abcam) and Vascular Endothelial- Cadherin (VE-Cadherin, Santa Cruz) were perfused into the device, incubated for 3 h, washed, and visualized with a goat-anti-mouse or anti-rabbit fluorescent IgG (Invitrogen) incubated for 3 h.

### Blood samples and human subjects

Human blood (Research Blood Components, Cambridge, MA), acquired in 3.2 % sodium citrate tubes was used within 5 h of blood draw, to prevent pre-analytical effects on platelet function. (Cattaneo et al. 2009). Institutional review board (IRB) approval was obtained for use of discarded blood samples. Subjects were selected from among patients who were taking antiplatelet medication. A total of 11 samples were used for analysis; of these, 8 subjects were on aspirin alone and 3 were on aspirin and clopidogrel (Plavix).

### Blood perfusion

The inlet reservoir of the microfluidic device was filled with whole blood (500 μL), containing platelets labeled with human CD41-PE antibody (10 μL/mL blood, 10 min incubation, Invitrogen), and sometimes containing fluorescently labeled fibrinogen (10 μg/mL; Life Sciences). Blood was pulled through the device (30 μL/min) via the syringe pump, resulting in an arteriole-like shear rate of 750 s^−1^ (Papaioannou and Stefanadis 2005). After 2 min, 100 mM calcium (CaCl_2_) and 75 mM magnesium (MgCl_2_) were added to the blood in the reservoir (1: 10 ratio) to restore coagulation-activated blood clotting (Jain et al. 2016; Neeves et al. 2014).

### Image acquisition and analysis

Platelets were visualized using time-lapse fluorescence imaging (20×, NA 0.4), and a time series of a 10-frame panorama (6 mm long × 0.665 mm wide) of the microchannel was recorded every 30 s. Measurements were made near the center of the device, close to 1 cm downstream of the blood flow inlet. The maximum intensity of the resulting image stack was projected along time, thresholded, segmented and cropped to the central 200 μm of the channel width for analysis. Platelet coverage was computed from the binary image as the ratio of bright pixels to the total number of pixels in the image.

### Light transmission aggregometry (LTA)

Blood samples were centrifuged at 290 *g* for 10 min (no brake applied) to collect platelet-rich plasma (PRP). To obtain a reference solution for each sample, PRP was centrifuged at high speed to pellet platelets (1000 g, 10 min) and collect platelet-poor plasma (PPP). LTA was performed at 37 °C under magnetic stirring using a Chrono-Log Corporation instrument and reagents (ADP 10 μM and collagen 2 μg/mL).

### Statistical analysis

All data are presented as mean ± standard error (SEM). Two-tailed *P* values were obtained from the statistical *t-*test or one way ANOVA using GraphPad Prism V6.

## Results and discussion

### Formation and evaluation of microfluidic devices

**Fig. 1:**
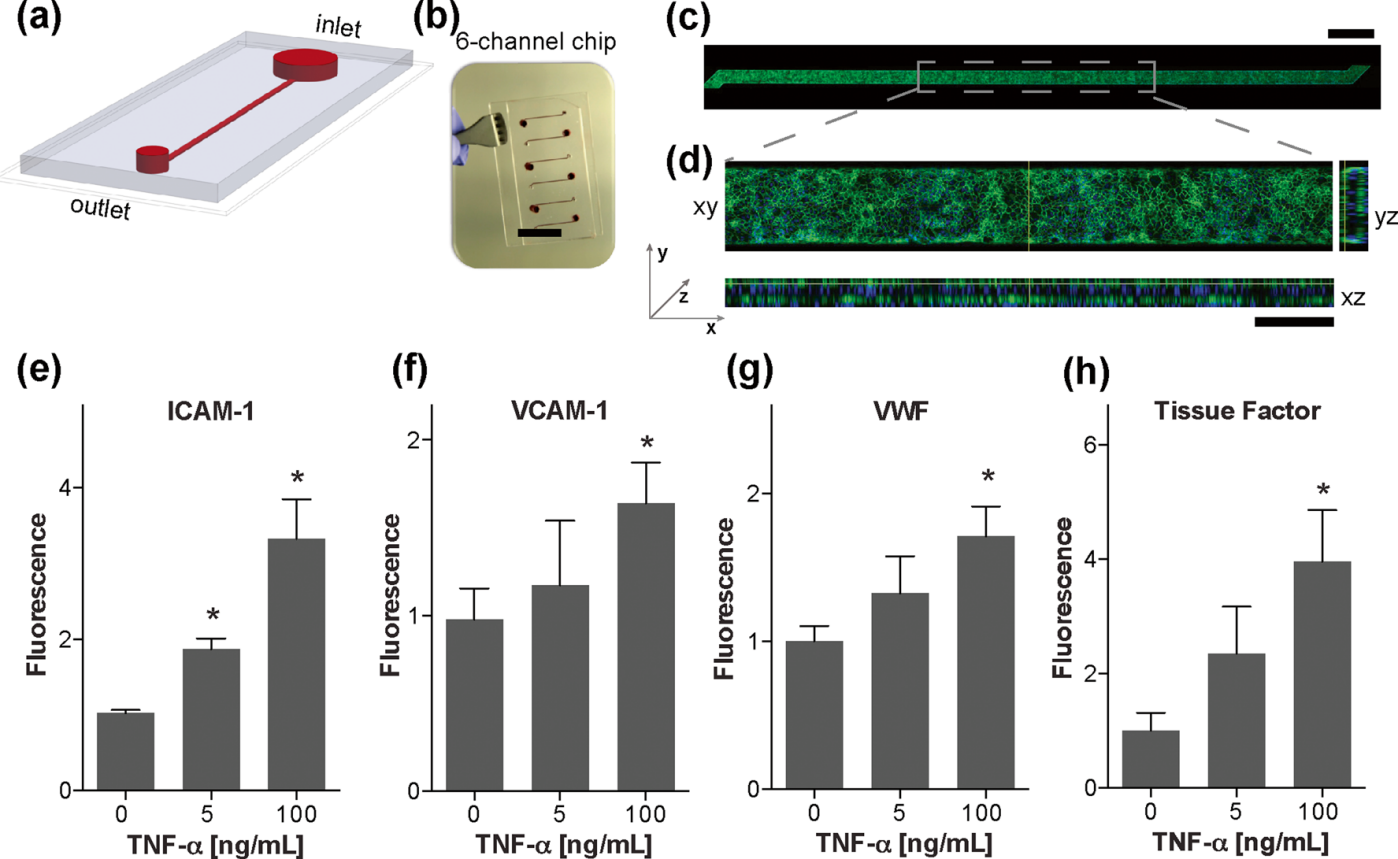
Engineering of the microfluidic device containing a chemically preserved endothelium. **a** Schematic of the microfluidic device showing a blood inlet reservoir, followed by the straight microchannel that ends at the outlet, where the syringe pump is attached to pull the blood, and **b** a photograph of a single polydimethylsiloxane (PDMS) microfluidic chip containing six independent microfluidic devices (bar, 15 mm) fabricated on a glass slide. **c** A fluorescence micrograph showing the entire microchannel covered with human umbilical vein endothelial cells (HUVECs) immunostained against the cell adhesion molecule, VE-cadherin (bar, 1 mm). **d** Confocal immunofluorescence microscopic images showing a section of the microchannel with HUVECs when viewed from above (‘xy’) and reconstruction of cross-sectional views from the front and the side (‘yz’ and ‘xz’, respectively) demonstrating full coverage of all microfluidic channel walls. (bottom; green, VE-Cadherin; blue, nuclear DAPI; bar, 200 μm). **e**–**h** Graph showing fluorescence (normalized by the untreated endothelium) measured after immunostaining the fixed endothelium with ICAM-1 (**e**), VCAM-1 (**f**), VWF (**g**), or tissue factor (**h**). *P < 0.05 versus untreated; *n* = 3

We used soft lithography to fabricate a microfluidic device containing a rectangular microchannel (400 μm wide, 100 μm high, 2 cm long, Fig. 1a), with six similar channels on each chip (Fig. 1b). The inner surface of all four walls of the microchannel were coated with collagen prior to culturing HUVECs on this surface to create a tube lined by a continuous, confluent endothelial cell monolayer (Fig. 1c, d, Movie 1). Multiple endothelial adhesion molecules are involved in the recruitment of blood cells and platelets during thrombosis *in vivo*. (Sagripanti and Carpi 2000) In previous studies, we showed that treatment of living endothelium cultured in microfluidic channels with the inflammatory cytokine TNF-α results in an increase in expression of surface adhesion molecules (e.g., ICAM-1, VCAM-1) within 4 h after addition (Benam et al. 2016; Huh et al. 2010). To explore whether a fixed endothelium would retain expression of surface molecules that could potentially exacerbate inflammation and thrombosis, we pre-activated the endothelium inside the device by adding increasing doses of TNF-α (0, 5, and 100 ng/mL) for approximately 18 h, and then fixed them with paraformaldehyde, rinsed three times and stored them at 4 °C in a humid environment. Interestingly, we found that the fixed endothelium continued to exhibit a dose-dependent increase in staining for ICAM-1 (Fig. 1e) and VCAM-1 (Fig. 1f), as well as VWF (Fig. 1g) and tissue factor (Fig. 1h), which mediate thrombus formation induced by TNF-α by promoting adhesion of blood cells and platelets (Sagripanti and Carpi 2000; Verhamme and Hoylaerts 2006; Wu and Thiagarajan 1996).

### Fibrin and platelet function analysis

**Fig. 2:**
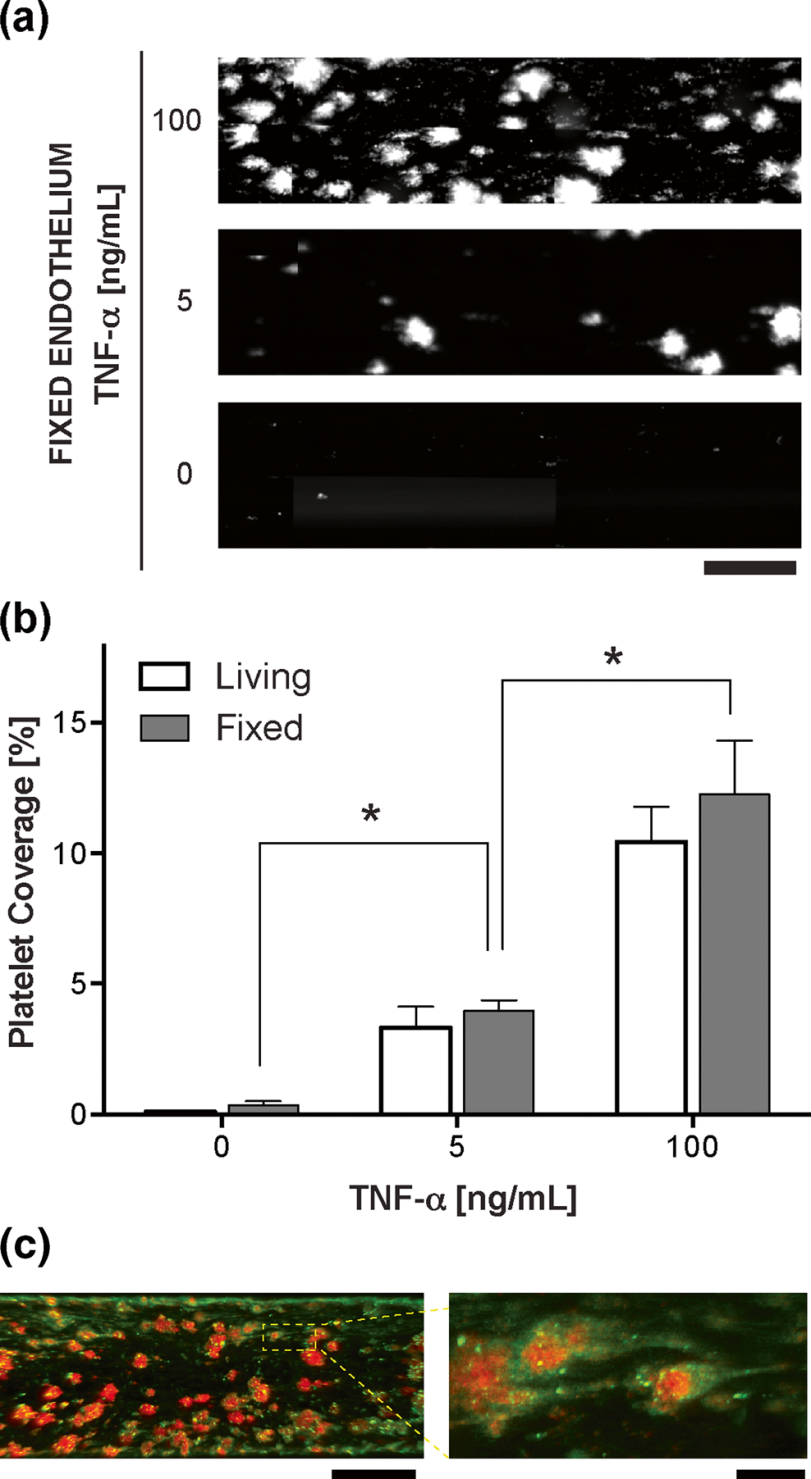
Platelet coverage and fibrin formation on the fixed endothelium in the microdevice. **a** Representative maximum intensity projection micrographs showing fluorescently labeled platelets adhering to the fixed endothelium in a tumor necrosis factor (TNF-α) dose-dependent manner (bar, 100 μm). **b** Graph showing platelet coverage when blood is perfused inside the microchannel lined with a living or fixed endothelium that was stimulated by TNF-α before fixation. Comparison of living vs. fixed microchannels was not significantly different (*P* > 0.05) at each TNF-α concentration. (*n* = 4; *P < 0.05). **c** Fluorescent micrograph shows fibrin (*green*) is formed along with platelet aggregates (*red*) on a fixed endothelium that was pretreated with TNF-α and perfused with recalcified citrated whole blood (left, bar, 200 μm; right, bar, 20 μm)

Next, we explored whether the fixed endothelium that had been stored for 24–36 h would retain its ability to promote haemostasis and thrombus formation when we perfused recalcified citrated whole blood through the device at an arterial shear rate of 750 s^−1^ (25 dyne/cm^2^), while analyzing platelet adhesion over 15 min of flow. When blood from a healthy donor was flowed over a fixed quiescent endothelium, there was virtually no platelet adhesion on the surface (Fig. 2a, b), consistent with the low level of expression of vascular adhesion molecules (Fig. 1e–h). In contrast, when devices were used with endothelium that was pretreated with increasing doses of TNF-α prior to fixation, a dose-dependent increase in platelet adhesion to the surface of the fixed endothelium was observed (Fig. 2a, b and Movie 2). Importantly, we did not observe any significant difference in platelet adhesion when we compared living versus chemopreserved endothelium, with or without treatment with TNF-α (Fig. 2b). Thus, these findings confirm that the fixed endothelium retains its pro-thrombotic activity and supports physiologically relevant levels of cytokine-induced platelet adhesion after fixation and storage. Furthermore, when we perfused whole blood containing fluorescently labeled fibrinogen, we found that the thrombi also contained a significant amount of fibrin if the endothelium was pre-treated with TNF-α before fixation (Fig. 2c), which confirmed that the fixed endothelial surface also retains its ability to activate the coagulation cascade. The morphology and adhesion pattern (Movie 2) of these thrombi (shape of a teardrop containing a core and a surrounding shell) (Stalker et al. 2014). Also was similar to that of clots formed on living endothelium *in vivo*, (Cooley 2011; Falati et al. 2002) and significantly different from those formed on bare collagen-coated flow chambers (shape of long tethers) (Colace et al. 2011) Taken together, these results demonstrate that the microfluidic device lined by fixed human endothelium is capable of reproducing physiologically relevant thrombus formation.

### Analysis of antiplatelet therapy

**Fig. 3:**
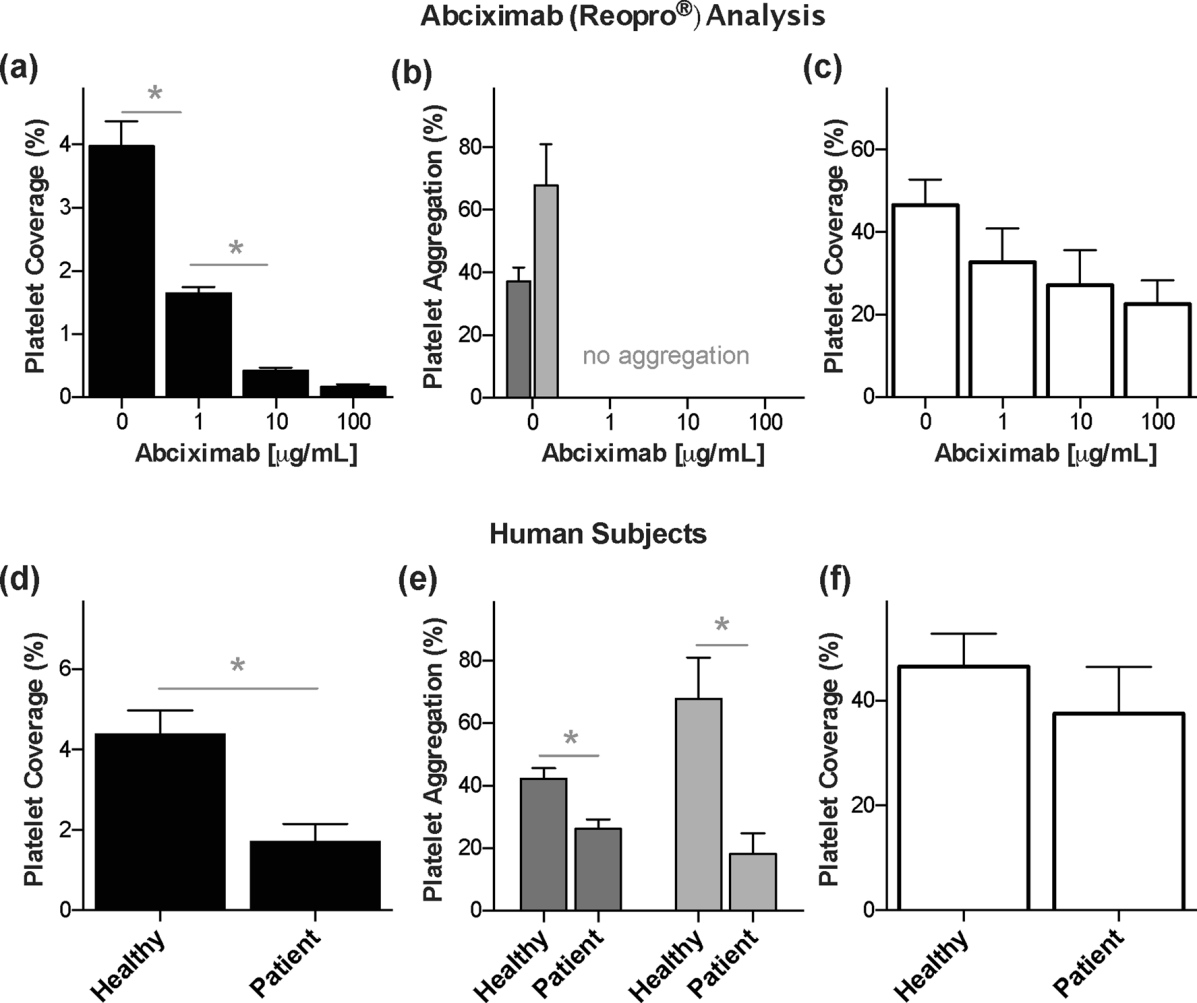
Assessment of antiplatelet therapy with the microfluidic device. **a** Platelet coverage on the fixed endothelium pretreated with TNF-α when blood samples containing different doses of the drug abciximab were perfused through the microfluidic device (*n* = 4) **b** Light transmission aggregometry of blood samples containing different doses of abciximab using either ADP  or collagen  as an agonist (*n* = 4). **c** Platelet coverage when blood samples containing different doses of the drug abciximab were perfused through collagen-coated microfluidic devices (*n* = 4). **d** Platelet coverage on the fixed endothelium pretreated with TNF-α when blood samples from healthy donors versus subjects treated with antiplatelet drugs were perfused through microfluidic devices (*n* = 11). **e** Light transmission aggregometry of healthy versus antiplatelet treated blood samples using ADP  or collagen  as an agonist (*n* = 11). **f** Platelet coverage when healthy versus subject blood samples were perfused through collagen-coated microfluidic devices (*n* = 11). *P < 0.05

These findings inspired us to investigate if the microfluidic device containing the fixed endothelium could have potential value as a clinical diagnostic device, and thus, we carried out preliminary studies to determine whether it can be used to detect antiplatelet drug effects in healthy donors and patients taking antiplatelet medication. We first perfused a microdevice containing fixed endothelium that was pretreated with a physiologically relevant dose of TNF-α (5 ng/mL) (Damas et al. 1989; Kim et al. 2014; Schlitt et al. 2004) with whole blood from a healthy donor containing 0 to 100 μg/mL (clinical range ~1–10 μg/mL) (Mascelli et al. 1998) of the antiplatelet GP IIb/IIIa antagonist, abciximab (ReoPro®). By applying widefield microscopy as a quick and easy way to quantitate antiplatelet effects, we observed dose-dependent inhibition of platelet adhesion to the surface of the fixed endothelium, with optimal effects being observed at 10 μg/mL or higher (Fig. 3a), which is consistent with previous studies using flow cytometric analysis (Rossi et al. 2001). In contrast, all concentrations of abciximab produced virtually complete inhibition of platelet aggregation (no dose dependence) as detected using conventional LTA, regardless of whether adenosine diphosphate (ADP) or collagen was used as an agonist (Fig. 3b). Moreover, while there appeared to be a small suppressive effect on platelet adhesion when the same blood samples were flowed through a collagen-coated flow chamber without an endothelial cell layer, the differences in platelet adhesion induced by different abciximab doses were not statistically significant (Fig. 3c). Thus, the microfluidic device containing the fixed endothelium provided a more robust measure of platelet function, with a higher dynamic response across a range of abciximab concentrations than these standard platelet function assays that are currently used in clinical laboratories. Notably, these findings also indicate that the fixed surface of the endothelium retains its ability to modulate platelet interactions via the GPIIb/IIIa pathway that is involved in multiple thrombotic and vascular processes, as this is the target of abciximab (Bombeli et al. 1998).

We then perfused whole blood isolated from human subjects who are regular users of antiplatelet drugs through the microfluidic device. As expected, patients taking antiplatelet drugs showed a significant reduction in platelet aggregation when tested using the device compared to healthy donors (Fig. 3d). While similar results were obtained using conventional LTA (Fig. 3e), our microfluidic assay did not require sample preparation or use of exogenous agonists. Our assay also required only 15 min to complete compared to the longer time (~2 h) often required for completion of the LTA assay. Most importantly, platelet inhibition could not be reliably detected in these subjects using a collagen-coated flow chamber, as there was no significant difference in platelet coverage between normal controls and patients receiving antiplatelet therapy in that assay (Fig. 3f). Taken together, these findings suggest that our microfluidic device containing fixed endothelium could potentially be applied as a platelet function test in clinical diagnostic laboratories as well as point-of-care settings in the future, although much more clinical validation is still clearly required.

## Conclusion

Since the simple microfluidic devices described here contain human endothelial cells that are chemically preserved by fixation, they can be stored, shipped and used whenever required, either in a laboratory setting or point-of-care settings. Our demonstration that this shear-inclusive whole blood assay can be used to evaluate platelet aggregation and inhibition with drugs in patient blood samples provides the proof-of-concept that this microfluidic device offers a way to detect physiologically important contributions of the endothelium and dynamic blood flow in the analysis of haemostasis and thrombosis, which are neglected by currently available devices. The fixed endothelium may have lost some of its live *in vivo* functions (e.g., release of bioactive messengers like nitric oxide), and the exact mechanism by which our surface promotes platelet adhesion and thrombosis is most likely multi-factorial and will require further characterization to understand. Also, the inflammation-led thrombus formation that we see in our device is mechanistically distinct from platelet aggregometry and collagen flow chamber studies and possibly represents a more complex system that more faithfully replicates *in vivo* conditions, relevant to blood coagulation and platelet function. Correspondingly, our results suggest that in the 15 min period during which we analyze blood flow, the fixed normal (unstimulated) endothelium retains its ability to prevent blood clotting, whereas when it is preactivated with TNF-α prior to fixation, the endothelium effectively promotes platelet adhesion and thrombosis *in vitro*. Importantly, evaluation of this type of propensity for whole blood thrombus formation in a diseased setting is not possible with aggregometry or collagen chambers. While the simpler assays may allow dissection of individual signaling components (e.g., collagen activation pathways), the results obtained so far with those assays correlate poorly with clinical outcomes (Dahlen et al. 2012) likely because thrombosis associated with most diseases is related more to inflammatory changes than to wall injury. In contrast, our system that more closely mimics *in vivo* pathophysiological conditions may be able to provide a better indicator of patient risk for ischemic events.

Recently, thrombus formation and platelet adhesion have been visualized when flowing blood contacts various homogeneous relevant molecular ligands *in vitro*, (de Witt et al. 2014) but none of these ligands induce formation of thrombi with a morphology that mimic clots observed on the endothelial surface in laser-induced vascular injury models *in vivo* (Cooley 2011; Falati et al. 2002), whereas our assay is able to reconstitute this response (Fig. 2a–c, Movie 2). Also, while we only demonstrated retention of expression of four prominent adhesive ligands (VWF, TF, ICAM-1 and VCAM-1) on the surface of the fixed endothelium, additional adhesion molecules (e.g., other integrins) could also be present, which could be explored in future mechanistic studies that are beyond this initial report describing the novel functional capabilities of this microfluidic device-based haemostasis and thrombosis assay. Nevertheless, our results show that this simple assay permits qualitative and quantitative analysis of pro-thrombotic and pro-coagulant responses of the fixed endothelium, and that the results we observed closely mimic those exhibited by living endothelium. Because we analyze clot formation in cytokine-stimulated endothelium, the measurements obtained using this device also might be more indicative for thrombus formation on an inflamed endothelium as might be found, for example, in an atherosclerotic plaque (Neeves et al. 2014), than those obtained with conventional hemostasis assays.

In this study, we cultured easily obtained HUVECs, but similar studies can be carried out using endothelium derived from primary cells or induced pluripotent (iPS) cells, which opens up the possibility of developing patient-specific diagnostics and advancing personalized medicine (Lian et al. 2010; Nelson and Terzic 2011). Importantly, given the small size, simplicity and robustness of this microdevice, it also could potentially be utilized for haemostasis and platelet function monitoring in point-of-care settings and for discovery of new platelet modulators or anti-inflammatory drugs.

## Electronic Supplementary Material

ESM 1 (AVI 5073 kb)

ESM 2 (AVI 533 kb)
